# Statins: HMG-CoA Reductase Inhibitors as Potential Anticancer Agents against Malignant Neoplasms in Women

**DOI:** 10.3390/ph13120422

**Published:** 2020-11-25

**Authors:** Anna Markowska, Michał Antoszczak, Janina Markowska, Adam Huczyński

**Affiliations:** 1 Department of Perinatology and Women’s Health, Poznań University of Medical Sciences, 60-535 Poznań, Poland; annamarkowska@vp.pl; 2 Department of Medical Chemistry, Faculty of Chemistry, Adam Mickiewicz University, 61–614 Poznań, Poland; michant@amu.edu.pl; 3 Department of Oncology, Gynecological Oncology, Poznań University of Medical Sciences, 60-569 Poznań, Poland; jmarkmed@poczta.onet.pl

**Keywords:** statin therapy, anticancer activity, malignant neoplasms, breast cancer, endometrial cancer, ovarian cancer, cancer prevention, adjuvant chemotherapy

## Abstract

Statins, also known as HMG-CoA inhibitors, are a class of bioactive small molecules that efficiently reduce the levels of cholesterol, and therefore are commonly used to manage and prevent various cardiovascular diseases. With respect to their original medical indications, statins are currently in the group of the most prescribed drugs worldwide. Of note is that statins are perceived actually rather as agents that have pleiotropic activities; in addition to their inhibitory activity on the production of endogenous cholesterol. Statins may also affect cell proliferation, angiogenesis and/or migration (metastasis) of different cancer cells, and play a positive role in the chemoprevention of cancer, thus being the excellent candidates to be repurposed in oncology. Particularly intriguing in this context seems to be the promising role of statins on both the incidence and course of common malignant neoplasms in women. In this article, we review and discuss the effect of the use of statins in the treatment of three types of cancer, i.e., breast, endometrial and ovarian cancer, with the highest mortality among gynecological cancers.

## 1. Introduction

**Figure 1:**
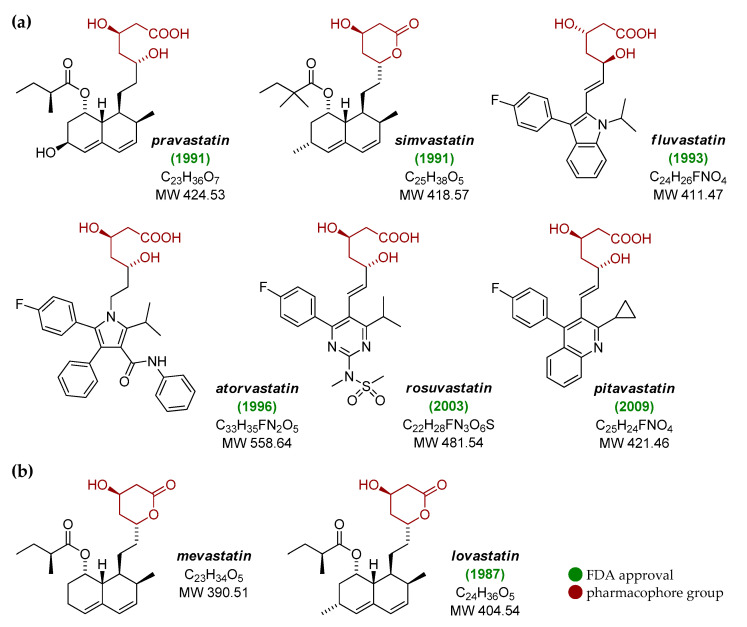
The structure of (**a**) six currently marketed statins, and (**b**) mevastatin (compactin) and lovastatin (mevinolin), the first two isolated and known statins. Both mevastatin and lovastatin are naturally-occurring statins, pravastatin is derived from mevastatin via biotransformation process, simvastatin is a semi-synthetic analog of lovastatin, while fluvastatin, atorvastatin, rosuvastatin and pitavastatin are fully synthetic compounds from the statins class.

Cardiovascular diseases (CVDs) are the main cause of death worldwide, responsible for more than 75% of all deaths in low- and middle-income countries. According to the World Health Organization, nearly 18 million people die every year because of the disorders of the heart as well as blood vessels, such as cerebrovascular, coronary heart and rheumatic heart diseases [1]. In the group of well-known factors that may initiate and further promote CVDs is a high plasma level of low density-lipoprotein (LDL) cholesterol [2]. With respect to the biosynthetic pathway of cholesterol, 3-hydroxy-3-methylglutaryl coenzyme A (HMG-CoA) reductase plays a pivotal role in the control of the rate limiting step in its production, and thus the inhibition of the activity of this crucial enzyme has been pinpointed as the prime target of effective cholesterol-lowering therapies [3]. In this context, the treatment strategies with the use of statins have been found to significantly reduce the elevated levels of atherogenic lipoproteins, primarily LDL cholesterol, mainly through the inhibition of HMG-CoA reductase [4]. Currently, six forms of statins are marketed and commonly used as the cholesterol-lowering drugs, leading finally to the reduction of both morbidity and mortality among patients with CVDs; in this group are atorvastatin, fluvastatin, pitavastatin, pravastatin, rosuvastatin, as well as simvastatin (Figure 1a). Several dual treatment strategies with the use of statins in the combination with other agents, like simvastatin/niacin extended-release, are also available [5].

**Figure 2:**
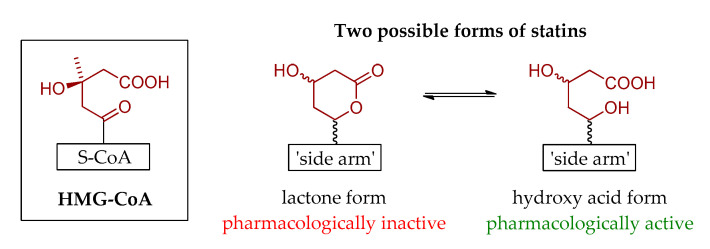
The similarity between structures of HMG-CoA molecule and statins.

Structurally, all statins have a common element (pharmacophore group) (Figure 1) that shows great similarity to the HMG-CoA molecule (Figure 2), thus being the excellent competitive inhibitor of HMG-CoA reductase [6]. With respect to the ‘side arms’ of most commercially available statins, they constitute partially hydrogenated naphthalene ring, with either the 2-methylbutyrate or 2,2-dimethylbutyrate moiety, and one or two methyl groups located on the opposite sites of the bicyclic ring system. On the other hand, the basis of the structure of synthetic statins is indole (fluvastatin), pyrimidine (rosuvastatin), pyrrole (atorvastatin) or quinoline (pitavastatin) aromatic ring substituted with various groups, including 4-fluorophenyl one, identical for all these structures. Nevertheless, as far as statins are concerned, the history of their isolation, synthesis and further development goes back nearly 50 years, and is briefly described in the next section.

## 2. Brief History of Statins

Before the discovery of the first statins, only a few lipid-lowering agents were known; they included cholestyramine, clofibrate and nicotinic acid [7]. However, as the activity of these compounds was rather moderate, development and introduction of more effective and less toxic inhibitors of cholesterogenesis was of top interest. In 1976, Endo and co-workers discovered compactin, renamed mevastatin (Figure 1b), a multifunctional fungal metabolite produced by *Penicillium citrinum* [8]; it was also later isolated from cultures of *Penicillium brevicompactum* [9]. After a series of subsequent animal experiments, this naturally-occurring compound has been shown to exhibit potent competitive inhibitory effects on the activity of HMG-CoA reductase and biosynthesis of cholesterol [10,11,12,13,14,15,16]. As mevastatin has been demonstrated to show therapeutic effects in humans by reducing the plasma levels of LDL cholesterol [17,18], but also some adverse side incidences, like lymphomas in dogs when using it in particularly high doses [7,19], intensive efforts have been made to identify other agents from this group of small bioactive molecules.

As a result of a screening program for HMG-CoA reductase inhibitors, lovastatin, known also as mevinolin (Figure 1b), was isolated from a mold *Aspergillus terreus* in the late 1970s [20], and then found to be more effective than mevastatin when tested in animal models; for review see [21]. Of note, a series of clinical trials have proven the ability of this compound to reduce the levels of both total and LDL cholesterol in patients with severe hypercholesterolemia [22,23,24,25,26,27,28]. The promising hypocholesterolemic effects of lovastatin led finally to its introduction to clinical therapy in 1987 as the first statin in history [3]. Since a natural lovastatin had been commercialized, six semi-synthetic and synthetic statins have been introduced to the markets in the last 30 years [7]. Among them were pravastatin and simvastatin (Figure 1a), the analogs of mevastatin and lovastatin, respectively, which have been found to effectively decrease the lipid profiles in a series of large and well-controlled clinical trials [29,30,31,32,33]. On the other hand, four synthetic variants of statins, fluvastatin, atorvastatin, rosuvastatin and pitavastatin (Figure 1a), were approved for routine medical practice in 1993, 1996, 2003 and 2009, respectively, thanks to which millions of patients around the world using these pharmaceuticals for primary prevention may have avoided heart attacks altogether [34,35].

**Figure 3:**
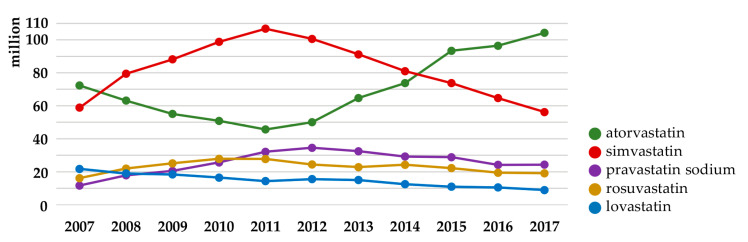
The number of prescriptions of statins in the United States in the period 2007 to 2017 (source: https://clincalc.com; accessed on 19 September 2020).

Statistically, in 2017, atorvastatin became the second most prescribed pharmaceutical in the United States, with more than 104 million prescriptions (Table 1) [36]. Although the total number of prescriptions of simvastatin has been systematically falling since 2011 (~110 million in 2011 *versus* ~60 million in 2017), the sale of pravastatin, rosuvastatin and lovastatin remains almost at the same level up to now (Figure 3). Of note, it was estimated that more than one billion people globally would qualify to be prescribed a drug from the statins class [37].

In addition to the original medical indications of statins, some interesting studies have suggested recently that selected compounds from this group may also play a positive role in cancer prevention [38,39], thus they can be considered as promising repurposable oncological drug candidates. Drug repurposing is a relatively novel strategy for the identification of possible alternative uses of well-known medications which are outside the scope of their original therapeutic indications. Importantly, such a strategy could entail a number of advantages compared to standard *de novo* drug development, such as less time-consuming and reduced costs of introduction of a drug to the market [40,41].

In this context, in the following paragraphs, the effects of statins on three malignant cancers occurring in women are discussed; these are the breast, endometrial and ovarian cancer, with the highest mortality among gynecological types of neoplasm. Although the results seem to be slightly ambiguous, the positive role of statins on both the incidence and course of these selected types of cancer, as well as their possible chemopreventive action is worth the attention.

## 3. Anticancer Activity of Statins

### 3.1. Breast Cancer

Breast cancer (BC) is the most common malignant neoplasm in women. In 2018, more than two million women were diagnosed with this type of cancer, which caused about 630,000 deaths; it accounts for 24% of all cases and 15% of total deaths from all malignant neoplasms in women, respectively [42]. The majority of cases occur in women aged 50–70, and the risk factors for the development of this cancer include, but are not limited to, mutations in genes (breast cancer 1 and 2, BRCA1/2) and the influence of hormones (contraception, hormone replacement therapy). Approximately 10–20% of BCs are a heterogeneous group of triple negative BCs (TNBCs), with an earlier age of development, aggressive course of the disease, lack of progesterone and estrogen receptors (ER^–^), and expression for human epidermal growth factor receptor 2 (HER2) [43]. Importantly, the risk of BC development in postmenopausal women is associated with excess weight and obesity, which causes *i.a.* changes in circulating cholesterol levels and pro-inflammatory cytokine levels. Statins are frequently prescribed as part of women’s long-term treatment programs. There is a need therefore, for further research into the effects of statins from the perspective of cancer prevention and treatment [44,45].

The studies by Islam and co-workers [46], based on electronic databases of 121,399 patients, did not show a clear relationship between statin use and the risk of BC development (RR = 0.94, 95% CI 0.86–1.03). Similar results showing that statin treatment does not influence the progress of BC were obtained by the researchers of the Nurses’ Health Study (NHS) [47]. Very briefly, more than 79,518 postmenopausal women participated in the survey based on questionnaires (confirmed by a physician). The study covered the period from 2000 to 2012, and 3055 women with invasive BC were qualified for the analysis, of which over 1900 individuals had never used the statin-based therapy. Ductal carcinoma was diagnosed in most of the patients (1952 cases), in some lobular carcinoma (313 cases), while in the rest of the participants the carcinoma in situ. In addition, some cases of ER^−^, but also estrogen receptor positive (ER^+^) carcinomas were determined. Of note, statin use was neither associated with the risk of infiltrating lobular carcinoma, ductal cancer nor estrogen receptor status (HR = 0.96, 95% CI 0.82–1.1 and HR = 1.1, 95% CI 0.92–1.3, respectively). According to the authors of this article, future analyzes need to take into account the type of statins used and the specific histological subtype of BC.

Metabolic syndrome occurs in some women with BC, and in this context the effect of the antidiabetic metformin and statins is frequently analyzed. According to data from the Finnish national diabetes database, 2300 women were diagnosed with BC, and then were treated with metformin, insulin and statins. Case-control analysis clearly showed that there was no difference in cancer incidence between the metformin group (HR = 1.02, 95% CI 0.93–1.11) and the statin group (HR = 0.97, 95% CI 0.89–1.05), while insulin treatment was associated with slightly increased morbidity (HR = 1.18, 95% CI 1.03–1.36) [48].

Furthermore, a study by Yao and co-workers [49] suggested that lipophilic statins, particularly lovastatin (Figure 1b), preferentially target TNBCs compared to other types of BC. The authors believe that the future therapy of TNBCs with statins should take into account the role of these compounds in cancer cells with stem-like phenotype (cancer stem cells, CSCs), develop the possible use of nanoparticles to encapsulate statins, and identify the molecular targets and mechanisms underlying such promising effects. The results of a study by the Danish Breast Cancer Group covering the period 2007–2017, with participation of over 14,000 women, also demonstrated the beneficial effects of the statin-based treatment option. In a multivariate analysis, statin treatment reduced the recurrence rate among women with stage I–III BC with the presence of estrogen receptors treated with aromatase inhibitors (HR = 0.72, 95% CI 0.50–1.04), suggesting that statins may become an additional therapeutic regimen in future [50]. As BC patients are exposed to cardiotoxic therapies—such as anthracyclines and trastuzumab—statins could be included in the BC treatment process for their cardioprotective effect [51,52].

Finally, the use of statins reducing death in BC patients has been shown to be strictly associated with the reduction of metastases; Beckwitt and co-workers [53] observed in their studies the inhibitory effect of atorvastatin (Figure 1a) on the proliferation of metastatic foci on BC cell lines that metastasize to the liver and lungs. Clinical observations indicate that both BC metastases and relapses are a sign of a poor course of the disease, and in this context some recent studies suggest that lipophilic statins can prevent recurrence of BC [54]. However, it is most likely not associated with variants of genes encoding drug transporters and simvastatin (Figure 1a) metabolizing enzymes (CYP enzyme group) [55].

### 3.2. Endometrial Cancer

On the basis of the world epidemiological statistics, endometrial cancer (EC) is a common malignant neoplasm in women. It ranks 6th as the cause of death after BC, colorectal cancer, lung cancer, cervical cancer and thyroid cancer. In 2018, over 380,000 new cases of EC were diagnosed, which accounted for 4.4% of all cancer cases in women [42]. There are two known types of EC: (i) type I EC, histologically endometrioid, which accounts for about 80% of all ECs, has a good prognosis, and is associated with well-known risk factors, like metabolic syndrome (diabetes, hypertension, obesity), mutations in the mismatch repair (MMR), phosphatidylinositol-4,5-bisphosphate 3-kinase catalytic subunit α (PIK3CA), Kirsten rat sarcoma viral oncogene homolog (KRAS), β1-catenin (CTNNB1) genes, and microsatellite instability (MSI), as well as (ii) non-endometrioid, less common type II EC, which has aggressive clinical course, and is often characterized by mutations in tumor protein p53 (TP53), HER2/neu and BRCA [56,57]. Although genomic analysis divides EC into four subgroups, there is an association of some EC subtypes with body weight (body mass index, BMI), which clearly suggests a modelling effect of obesity on the genetic types of EC malignancy [58,59].

Numerous studies have shown hyperglycemia and type 2 diabetes increase the risk of developing EC, especially EC type I. Likewise, research indicates a reduction in morbidity, as well as a more favorable clinical course as a result of the application of drugs, such as metformin, which reduce these factors [58,59,60]. However, the results of the studies on the use of statins in EC remain inconclusive [61,62,63,64,65], and the observational studies often compare the effects of two drugs, i.e., antidiabetic metformin and a selected compound from the statins class [48,49].

The studies by Kim and co-workers [61] on established three cell lines, including the Ishikawa line, proved that both metformin and simvastatin (Figure 1a) used either separately or together in the experiments inhibit tumor growth and metastasis. These therapeutics mediated cell apoptosis, as determined by executive caspase 3 levels as well as Bax, Bcl-2 and Bim markers, but also inhibited the mammalian target of rapamycin (mTOR) signaling pathway strictly involved in cell proliferation, angiogenesis, and protein synthesis. On the other hand, a retrospective analysis of the Finnish Diabetes Registry, including 92,366 women with newly diagnosed type 2 diabetes and 590 with EC type I, has shown that statin treatment was inversely related to the frequency of EC in these individuals (HR = 0.78, 95% CI 0.65–0.94). Nevertheless, metformin and other antidiabetics had an unfavorable effect, increasing the incidence of EC compared to the group of women not treated for diabetes (HR = 1.24, 95% CI 1.02–1.51 and HR = 1.25, 95% CI 1.04–1.50, respectively) [64].

Interestingly, Yang and co-workers [65] presented a meta-analysis of 9517 women with EC diagnosed between 2001 and 2016 on different continents (North America, Europe and Asia) and treated with statins. These analyses, also based on electronic databases, included two randomized and 11 non-randomized studies (four cohorts and seven case-control studies), with a median duration of 5.2 years. According to the authors of this article, statin use was found to reduce the risk of developing EC only in the group of women from Asia (437 cases) (RR = 0.52, 95% CI 0.37–0.74). It is worth noting that, in this study, the use of cardiovascular drugs, such as aspirin, may have been a confounding factor, as noted by the researchers analysing the data.

Also on the basis of electronic databases, Liu and co-workers [66] analysed the risk of developing gynecological cancers in women using statins, including 10 studies with a total of 9957 patients with EC. Although it was suggested that statin treatment did not significantly reduce the risk of developing EC (RR = 0.90, 95% CI 0.75–1.07), the risk decreased (RR = 0.69, 95% CI 0.44–1.10) if statins were used for more than 5 years. Intriguingly, as previously observed by Yang and co-workers [65], Asian women using statins once again were at lower risk of developing EC than individuals from America or Europe (RR = 0.46, 95% CI 0.28–074) [66]. Contrary to these results, the PRISMA meta-analysis, based on the results of electronic databases of 19 studies that included 199,362 women and 19,849 cancer cases, including 11,901 EC cases, suggested not only that the use of statins reduced the risk of developing EC (RR = 0.88, 95% CI 0.78–1.00), but also revealed no association between long-term statin use (5 years) and a reduction in the risk of developing this type of cancer [62]. On the basis of the Danish Cancer Registry, Sperling and co-workers [67] analysed EC mortality in 6694 women with EC aged 38–84, using statins; type I EC was found in 5982 women (89% of all ECs) and type II in 712 women (11%). Of note is that post-diagnosis statin use was found to be associated with lower mortality (reduced by even 39%) among patients compared to non-statin users (HR = 0.61, 95% CI 0.48–0.77). This relationship was similar for EC I and II type (HR = 0.59, 95% CI 0.45–0.77 and HR = 0.65, 95% CI 0.43–0.99, respectively). The mortality rate in women using statins before diagnosis was also lower, but not as pronounced as in those who used them post diagnosis.

Slightly different results have been presented by Li and co-workers [63]. Using electronic databases, they analysed the data of 5923 EC women from Austria, Denmark, Israel, UK and USA. The use of statins prolonged both overall survival and disease specific survival (HR = 0.80, 95% CI 0.66–0.95 and HR = 0.69, 95% CI 0.61–0.79, respectively). Moreover, the authors of this article were the first to find a dependence of disease specific survival prolongation in type II EC (HR = 0.64, 95% CI 0.50–0.81), which is very aggressive and has a poor prognosis due to relapses even in the early clinical stages. On the other hand, a group of Israeli researchers analysed the cases of women in 11 hospital centres from 2002–2014, with a mean follow-up of 6.2 years. Surprisingly, the 5-year relapse-free survival did not differ between the group of women with EC who had used statins before diagnosis (633 women, 32.8% of all respondents) and those who had not used them at all (1354 women, 67.1%). The 5-year relapse-free survival was 82% and 83% (*p* = 0.508), respectively, and the overall survival was 77% and 75% (*p* = 0.901), respectively [68]. The presented results regarding the impact of statin treatment on the incidence of EC and the course of this cancer seems thus far rather inconclusive. The proportion of presented results suggesting a beneficial effect of statins on the risk of developing EC and its course seems a promising development.

### 3.3. Ovarian Cancer

Ovarian cancer (OC) ranks as the 8th cause of death in women with all malignant neoplasms. In 2018, 295,414 women worldwide were diagnosed with OC, which accounts for 3.4% of all cancer cases [42]. Of note, this heterogeneous group of cancers (genetically determined and sporadic) is the leading cause of mortality among all gynecological cancers, with high-grade serous OC diagnosed in high clinical stages, often with relapses, which displays progressive resistance to platinum derivatives, and thus has a poor prognosis [69].

Similar to BC and EC, the results of the studies on the influence of statins on the development of OC are also inconsistent. In the aforementioned PRISMA meta-analysis, Wang and co-workers [62] presented the results of 10 studies aimed at assessment of the influence of statins on the development of OC. On the basis of the calculations, it was shown that among 7948 cases of OC in women from North America or Europe, statin use did not significantly reduce the risk of developing this type of cancer (RR = 0.88, 95% CI 0.76–1.03). There was also no association between long-term statin use (>5 years) and the risk of developing OC (RR = 0.73, 95% CI 0.51–1.04). A similar lack of relationship between the statins treatment and development of OC was also shown by the Finnish Registry study in a group of women aged over 40 and diagnosed with type 2 diabetes. Among the cohort of 137,643 individuals studied in 1996–2011, 303 cases of OC were diagnosed. It was also the first study in women with type 2 diabetes treated with either metformin or insulin as well as statins, due to the increased risk of cardiovascular diseases and/or hypercholesterolemia. In a full cohort analysis, there was no evidence that metformin or insulin use was associated with a different incidence of OC compared to other oral antidiabetic agents. Furthermore, there was also no effect of statin use on the development of OC (HR = 0.99, 95% CI 0.78–1.25), and no interaction of statin treatment with metformin [70].

However, some other authors have reported a lower risk of developing OC in women using statins. Five studies have been based on the electronic database analyses for 624 women who had developed OC, from North America, Asia and Europe. Statin use was associated with a 21% reduction in the risk of developing this type of cancer without taking into account their heterogeneity (RR = 0.79, 95% CI 0.64–0.98). There was also no difference in the geographical subgroups of women (Asia or other continents) [66]. A systematic review and meta-analysis by Irvin and co-workers [71] in nine studies involving 435,237 women suggested that statin use was associated with a lower risk of developing OC. However, this risk depended on the OC histotype, the class of statins used and the duration of their use. The risk for OC without histotype was reduced (RR = 0.87, 95% CI 0.77–1.03), especially in women with low pravastatin (Figure 1a) treatment (RR = 0.83, 95% CI 0.70–0.99). The risk of developing serous carcinomas was reduced in relation to clear cell carcinomas (RR = 0.95, 95% CI 0.69–1.20 and RR = 1.17, 95% CI 0.54–1.10, respectively). According to the authors of this article, such dependencies on the cancer histotype, type of statins and their dose require further clarification.

The differences in the incidence of OC associated with the use of statins are partly explained by genetic studies on the inhibition of HMG-CoA reductase, which is affected by statins which lower the synthesis of endogenous cholesterol [72]. The GWAS (genome association study) meta-analyses from the Ovarian Cancer Association Consortium, involving more than 22,000 patients with invasive OC, as well as retrospective cohort studies of the Consortium of Investigators of Modifiers performed among BRCA1/2 mutation carriers, involving more than 3,880 women, have shown a clear relationship between LDL cholesterol resulting from inhibition of HMG-CoA reductase and reduction in the risk of developing OC (OR = 60, 95% CI 0.43–0.83). Importantly, in BRCA1/2 mutation carriers, the risk of developing OC was also reduced (RR = 0.69, 95% CI 0.51–0.93). On the other hand, the explanation of the beneficial effects of statins on inhibiting the development of OC is based, according to Liu and co-workers [73], on the activation of the c-Jun *N*-terminal kinase (JNK) signaling pathway and the reduction of the proapoptotic Bim protein. According to the authors of this article, such a molecular mechanism of action of statins inducing apoptosis in OC cells could lead to novel therapies for advanced OC.

The effect of statin treatment on OC survival has also been assessed in several studies. In a meta-analysis based on an electronic database of 12 studies, an improvement in overall survival was found in women using statins (HR = 0.76, 95% CI 0.68–0.85). This study was a part of a larger meta-analysis (36 studies) that included a group of women who were also treated with other drugs, such as metformin, β-blockers, aspirin or non-steroidal anti-inflammatory drugs (NSAIDs); neither drug has shown an overall survival benefit [74]. Jeong and co-workers [75] have reported results of a study (16 articles) of the influence of statins on survival and mortality of patients treated for various malignant neoplasms, including OC. From among the three meta-analyses of OC, one suggested a beneficial effect in reducing cancer-specific mortality (RR = 0.74, 95% CI 0.63–0.87). Favorable results concerning survival in OC following from the data for more than 2,100 patients with this type of cancer have been also documented by Harding and co-workers [76]. In the group of over 2,100 women with OC (≥66 years of age), statin use was associated with a lower risk of death from cancer (RR = 0.74, 95% CI 0.60–0.91). Similarly, Couttenier and co-workers [77] in a retrospective study of the data from the Belgian Cancer Registry for over 5400 OC patients found that the use of statins, especially simvastatin and rosuvastatin (Figure 1a), after cancer diagnosis prolonged survival (HR = 0.86, 95% CI 0.74–0.99 and HR = 0.71, 95% CI 0.55–0.92, respectively).

The positive effect of statins used in OC patients may be explained by the fact that serous OC with the highest malignancy exhibit specific oxidative metabolism responsible for the inflammatory response and drug resistance. Research by Criscuolo and co-workers [78] revealed that platinum-resistant OC cells show reduced biosynthesis of cholesterol, mainly due to decreased activity of the enzymes involved in this process, and they capture exogenous cholesterol for their needs. With regard to this metabolism, a dual mechanism of statins action has been demonstrated – on the one hand, when used to inhibit cholesterol biosynthesis, they reduce cisplatin-induced apoptosis, but after silencing the enzyme (lipase G, LIPG) involved in lipid metabolism as well as removing lipids from the culture medium, they increase the sensitivity to platinum drugs. These results raise concerns about the use of statins in OC patients and point to the necessity of taking into account lipid metabolism in the treatment.

In connection with the above described results, an article trying to respond to the question "How much of it is true?" has been published [79]. The author of this paper hypothesized that statin therapy could improve the survival of women with OC. The mechanism of this action in OC is based on inhibitory interaction with the signal transducer and activator of transcription 3 (STAT3) signaling pathway, which is activated by the cytokine interleukin 6 (IL-6); IL-6 can be induced in response to pro-inflammatory interleukin 1β (IL-1β), whose elevated levels are seen in OC. Thus, statins not only lower cholesterol, but also suppress inflammation. Further research is however necessary.

## 4. Conclusions

Statins, HMG-CoA reductase inhibitors commonly used as cholesterol-lowering therapeutics, represent one of the most commonly prescribed classes of drugs worldwide. Six statins are currently marketed, i.e., atorvastatin, fluvastatin, pitavastatin, pravastatin, rosuvastatin, as well as simvastatin (Figure 1a). Interestingly, some recent findings also suggest the positive role of statin-based regimens in reducing the incidences of non-cardiac mortality, particularly those linked with cancer.

The beneficial effect of statin use on the incidence and course of common malignant neoplasms in various localisations, histological subtypes, advancement in women is ambiguous. This is possibly due to the use of different types of statins and/or heterogeneous populations, dosage and duration of treatment. While some meta-analyses from electronic databases did not show the effect of statins on reducing the incidence of breast cancer, a series of other studies strongly suggested the positive impact of the use of these bioactive small molecules on both reduction in the recurrence rate and in the risk of death from breast cancer; similar results were observed for endometrial and ovarian cancer patients. The studies performed using established cancer cell lines were very promising; statins inhibited i.a. the mTOR signaling pathway strictly associated with cancer progression, and pro-inflammatory cytokine activity.

To sum up, in our opinion, statins should only be considered as an adjuvant therapeutic option in cancer patients with the internal medicine indications, such as hypercholesterolemia, metabolic syndrome or the prevention of circulatory disturbances in the cardiovascular and cerebral systems. Moreover, their use seems to be beneficial due to the cardioprotective effects in cancer patients treated with cardiotoxic therapies. Nevertheless, future studies, including large and well-controlled clinical trials, considering i.a. the type of statin used (hydrophilic or lipophilic), time of statin use (prediagnosis or postdiagnosis) as well as duration of treatment, are vital to validate correctly the promising effects of the use of statins in women with selected malignant neoplasms.

## Figures and Tables

**Table 1:** A summary of basic information about the most prescribed compounds from the statins class (source: https://clincalc.com; accessed on 19 September 2020).

Statin	Brand Name Synonyms	FDA Approval	Total Prescriptions (2017)	Rank/Change
atorvastatin	Lipitor^®^	1996	104,774,006	2/ 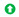 1
simvastatin	Flolipid^®^, Zocor^®^	1991	56,708,617	8/ 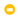 0
pravastatin ^1^	Pravachol^®^	1991	24,812,698	24/ 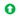 3
rosuvastatin	Crestor^®^, Ezallor^®^	2003	19,628,897	39/ 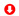 2
lovastatin	Altoprev^®^, Mevacor^®^	1987	9,453,815	84/ 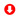 12

^1^ Pravastatin sodium.
